# Heart rhythm *in vitro*: measuring stem cell-derived pacemaker cells on microelectrode arrays

**DOI:** 10.3389/fcvm.2024.1200786

**Published:** 2024-02-21

**Authors:** Sophie Kussauer, Patrick Dilk, Moustafa Elleisy, Claudia Michaelis, Sarina Lichtwark, Christian Rimmbach, Robert David, Julia Jung

**Affiliations:** ^1^ Department of Cardiac Surgery, Rostock University Medical Centre, Rostock, Germany; ^2^ Department of Life, Light, & Matter, University of Rostock, Rostock, Germany

**Keywords:** microelectrode array, pacemaker cells, cardiomyocytes, *in vitro* drug testing, conduction velocity, embryoid bodies

## Abstract

**Background:**

Cardiac arrhythmias have markedly increased in recent decades, highlighting the urgent need for appropriate test systems to evaluate the efficacy and safety of new pharmaceuticals and the potential side effects of established drugs.

**Methods:**

The Microelectrode Array (MEA) system may be a suitable option, as it provides both real-time and non-invasive monitoring of cellular networks of spontaneously active cells. However, there is currently no commercially available cell source to apply this technology in the context of the cardiac conduction system (CCS). In response to this problem, our group has previously developed a protocol for the generation of pure functional cardiac pacemaker cells from mouse embryonic stem cells (ESCs). In addition, we compared the hanging drop method, which was previously utilized, with spherical plate-derived embryoid bodies (EBs) and the pacemaker cells that are differentiated from these.

**Results:**

We described the application of these pacemaker cells on the MEA platform, which required a number of crucial optimization steps in terms of coating, dissociation, and cell density. As a result, we were able to generate a monolayer of pure pacemaker cells on an MEA surface that is viable and electromechanically active for weeks. Furthermore, we introduced spherical plates as a convenient and scalable method to be applied for the production of induced sinoatrial bodies.

**Conclusion:**

We provide a tool to transfer modeling and analysis of cardiac rhythm diseases to the cell culture dish. Our system allows answering CCS-related queries within a cellular network, both under baseline conditions and post-drug exposure in a reliable and affordable manner. Ultimately, our approach may provide valuable guidance not only for cardiac pacemaker cells but also for the generation of an MEA test platform using other sensitive non-proliferating cell types.

## Introduction

Cardiovascular disease (CVD) remains the leading cause of death worldwide (1), according to the World Health Organization (WHO) (2). CVDs involve defects in both the vascular system and the heart. Therefore, pathologies may include vascular obstruction, structural abnormalities, and electrophysiological dysfunction. The latter can lead to life-threatening arrhythmias resulting from disorders of the conduction system, such as sinoatrial or atrioventricular block, or from structural abnormalities that give rise to ventricular tachycardia/fibrillation culminating in sudden cardiac death (3). Fortunately, many patients can receive effective treatment resulting in high survival rates. However, the healthcare system is experiencing an increase not only in the number of treatment-associated cardiac arrhythmias but also in the related costs (1). Due to their proarrhythmic risk or arrhythmia-inducing side effects, numerous drugs have either been withdrawn from the market or have not been approved (4–6). The Comprehensive *in vitro* Proarrhythmia Assay (CiPA) initiative and The Consortium for Safety Assessment using Human iPS cells (CSAHI) provide recommendations on how to analyze the proarrhythmic potential of established drugs and substances and how to evaluate drug tests (7–10). With regard to the withdrawal of potential drugs, the limited suitability of animal testing, given the detrimental transferability of experimental animal data to humans and its poor ethical justification, needs to be mentioned (11, 12). Therefore, it is necessary to evaluate *in vitro* drug testing methods capable of identifying potential cardiac side effects of developed drugs, especially on the physiological pacemaker.

The cardiac conduction system comprises the sinoatrial node (SAN), atrioventricular node, His bundle, bundle branches, and Purkinje fibers. Various cell- and gene-based methods have been used to develop biological pacemaker cells or other cells of the conduction system for clinical relevance, to decipher pathophysiological mechanisms or to model diseases *in vitro*. Initial attempts at SAN transplantation were conducted in the 1920s using tissue from the conduction systems. This progressed to more specific implantation of autologous SAN in the 1960s (13), and then to cell-based approaches involving only isolated SAN cells with electrical coupling (14–16). Alternatively, direct reprogramming of existing cardiomyocytes has been performed (17, 18). When using another cell source, such as ESC or iPSC, differentiated cardiomyocytes pose the problem of distinct subpopulations. This is because pacemaker cells make up no more than around 10%–20% of the cardiomyocyte population via *in vitro* generation, even though successful coupling and pacing can occur after implantation (19). However, specific pacemaker cell protocols have been developed over the last few years (20–27). Recently, three-dimensional applications of pacing cells have been carried out *in vitro* (28, 29). In addition to the sinoatrial node, other cells of the cardiac conduction system have been generated, such as atrioventricular node-like cells (30) or cell lines that help to decipher the respective structure (31). Recently, there has been a focus on Purkinje-like cell differentiation (32–34). Overall, those attempts have resulted in promising constructs that now display physiologically relevant heart rates and good autonomic responsiveness.

As indicated above, our group has established a protocol to generate functional pacemaker cells from PSCs via TBX3 programming, combined with antibiotic selection using the *α*Mhc-promoter (20, 35). By obtaining so-called “induced sinoatrial bodies” (iSABs) through our protocol, pacemaker cells with beating frequencies between 400 and 500 bpm were produced *in vitro.* This is the first time that these frequencies have been attained in cells that truly correspond to those found in a murine heart and *in vitro* cultured nodal cells that have been isolated from the mouse SAN (294 ± 59 min^−1^) (36).

Microelectrode Arrays (MEAs), which measure the extracellular field potential and provide information on electrophysiological properties, have been assessed as a suitable, real-time, non-invasive, and user-friendly platform for measuring and analyzing our cells (37). In cardiac risk assessment, an increasing number of studies have benefited from the advantages of the MEA technology, such as the ability to measure many cells in a syncytium, perform long-term experiments, and perform high-throughput screening (Multiwell MEAs) (7, 9, 10, 38–40).

However, it is important to adapt each measurement platform according to cell type-specific requirements such as coating, cell density, and enzymatic dissociation. The two-dimensional cultivation of pacemaker cells on the MEA requires the formation of proper extracellular attachment to the surface in order to increase the number of active electrodes that can be measured. Only good sealing can ensure the detection of the full range of field potentials. For instance, a coating of extracellular matrix proteins provides crucial binding sites for cellular adhesion proteins. Moreover, achieving an adequate number of cells is essential for the development of a synchronized syncytium through intercellular connections, which allows accurate measurement of the extracellular field potential.

## Methods

### Generation of pacemaker cell clusters

The generation of functional pacemaker cells from PSC has been described previously (20, 35). Briefly, programming murine ESC with TBX3 combined with antibiotic selection using the *α*Mhc promoter led to small aggregates that we termed “induced sinoatrial bodies” (iSABs).

Double-transfected PS cells (TBX3 and *α*Mhc promoter) were cultured at 37°C and 5% CO_2_ for at least 7 days before differentiation was initiated. The culture medium consisted of Dulbecco's Modified Eagle's Medium—high glucose with stable L-Glutamine (4 mM) (GIBCO, Life Technologies, Carlsbad, USA), 10% FCS superior (Biochrome AG, Berlin, Germany), 1% Penicillin-Streptomycin (GIBCO, Life Technologies, Carlsbad, USA), 100 µM MEM Non-essential amino acids (GIBCO, Life Technologies, Carlsbad, USA), 1,000 U/ml Leukemia inhibitor factor (diluted in ddH_2_O with 0.1% BSA) (Phoenix Europe, Mannheim, Germany) and 100 µM ß-mercaptoethanol (Sigma, St.Louis, USA). Subsequent selection of double-transfected cells was performed by adding 10 µg/ml blasticidin (InvivoGen, San Diego, USA) and 250 µg/ml hygromycin (InvivoGen, San Diego, USA).

To obtain iSABs, the first step was to induce differentiation using Embryoid Bodies (EBs). Consequently, *Hanging Drops* (HD) were generated, each containing approximately 400 cells. After 2 or 3 days in HD, the EBs were transferred to 10 cm petri dishes and maintained as a suspension culture for a further 3 days at 37°C and 5% CO_2_. On day 7 of differentiation, the EBs were plated on 0.1% gelatin-coated petri dishes with medium changes every 2–3 days. The EBs began to adhere, grow, and contract spontaneously. On day 15 of differentiation, the antibiotic-based selection of *α*Mhc-expressing cells was started using medium containing 400 µg/ml of G418 (Biochrome, AG, Berlin, Germany) and continued until treatment with collagenase IV (6,000 U/ml, GIBCO, Life Technologies, Carlsbad, USA). The latter was performed on day 20 of differentiation by incubating the cell layer with the enzyme solution for 8 min at 37°C. Mechanical agitation during pipetting separated the cell clusters from the cell layer. After centrifugation, the obtained iSABs were resuspended in a differentiation medium without ascorbic acid and then transferred to new petri dishes. The iSABs were subsequently separated from dead cells by filtration using a 40 µm cell strainer (EASystrainer, Greiner-BioOne, Frickenhausen, Germany).

The differentiation medium consisted of IMDM medium (PAN-Biotech GmbH, Aidenbach, Germany/Biochrom), 10% FCS superior (Biochrome AG, Berlin, Germany), 1% Penicillin-Streptomycin, 100 µM MEM Non-essential amino acids, 450 µM 1-thioglycerol and 1.21 µM ascorbic acid.

### Scale-up of EB generation in *sphericalplate 5d*

In order to reduce the time and space required for differentiation and to increase the yield of differentiated cells, we introduced a new method for EB formation into our existing protocol and analyzed the comparability of the generated pacemaker cells. Therefore, we compared the standard HD method with the spherical multiwell plate (*Sphericalplate 5D*, Kugelmeiers, Switzerland) (SP). The latter cell culture plate contains 12 out of 24 wells with 750 conical cavities each. Initially, the wells underwent incubation with pre-warmed PBS to minimize air bubbles when adding the cell suspension. For the *sphericalplate*, a cell suspension of 400 cells * 750 microwells was formulated for each individual well.

The EB diameter was measured after different generation times using the two mentioned EB-generating methods. Four independent experiments were conducted with 10 EBs measured per condition. We analyzed the diameter of the EBs after one, two, or three days of generation. After the appropriate time, the EBs were harvested and collected by gently washing them through the wells (SP) or plates (HD). The diameter of the EBs was then measured bidirectionally using a Zeiss Elyra PS1 microscope.

Moreover, the average number of beating foci per EB was analyzed by calculating data from 10 adherent EBs. Briefly, after formation using HD or SP, 10 EBs were either plated directly onto gelatin-coated wells (24 well plates) or cultured as “swimming” non-adherent EBs in uncoated petri dishes for an additional 3–4 days and then plated. At days 16/17 of differentiation, the number of beating areas per EB was microscopically detected and measured using the Zeiss Elyra PS1.

### Beating frequency analysis

Following various induction conditions, cells underwent differentiation, pacemaker cells were selected, collagease treatment and filtration were performed. Afterwards, gained iSABs clusters were moved to 6 cm petri dishes using differentiation medium without ascorbic acid. The beating frequency was evaluated 3–6 days after collagenase treatment utilizing an ELYRA PS.1 LSM 780 microscope. Video recordings of the beating areas were captured using Zen Black software (Zeiss, Jena, Germany) at a sampling rate of 20 frames per second. The exposure time was set to 50 ms and the recording duration was 30 s for each 512 × 512 pixel frame. The microscope incubator temperature was maintained at 37°C during imaging.

**Figure 1:**
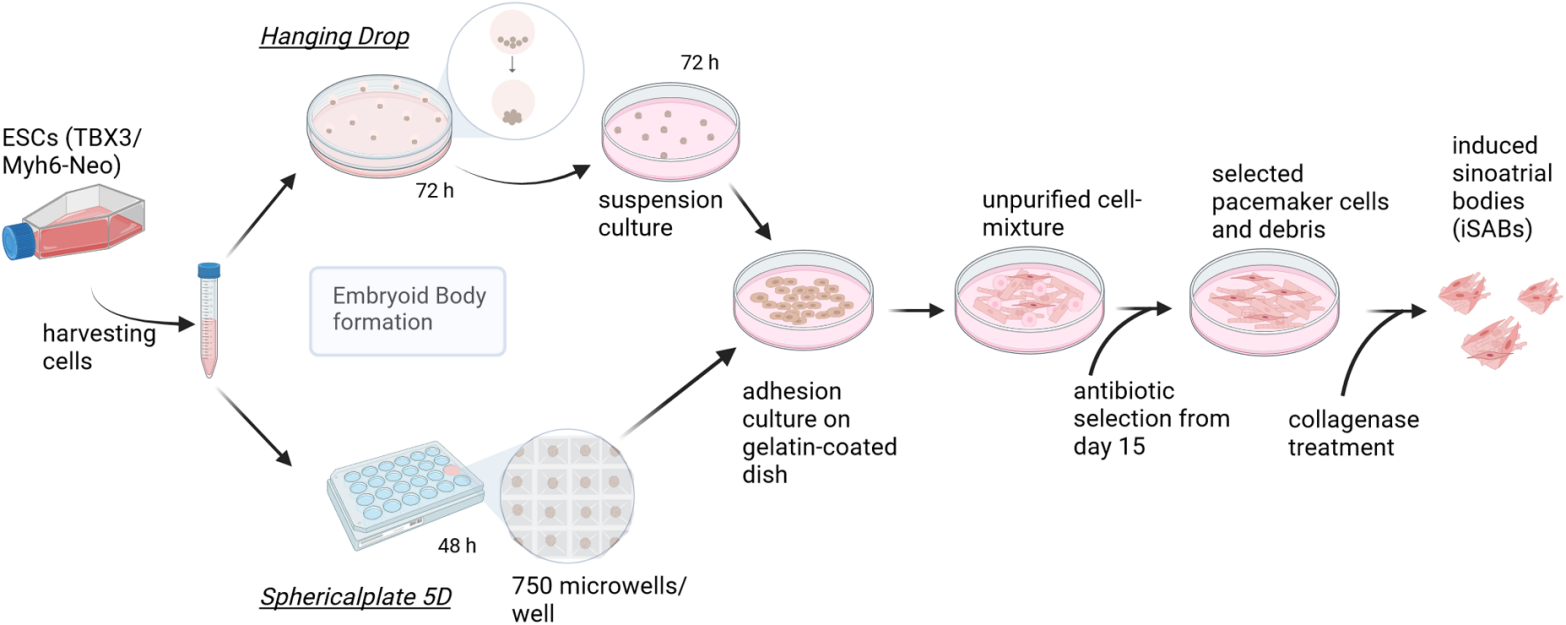
Pacemaker cell differentiation process—methodological comparison of embryoid body generation techniques. Graphic summary of the final differentiation procedure, which starts by splitting the cells creating EBs of approximately 400 cells, using either hanging drops (HD) (according to Rimmbach et al. (35) or spherical plates (SP) and subsequent seeding. This is followed by selection based on antibiotics and the formation of induced sinoatrial body (iSAB) clusters via collagenase treatment. The hanging drop method yields a maximum of 144 EBs per square petri dish, whereas one well of the *sphericalplate* generates 750 EBs. Created with BioRender.com.

Considering the previous results of EB diameter and beating frequency, hanging drops, hanging for 3 days, and a spherical plate, lasting for 2 days, were chosen for all further experiments. The final pacemaker cell differentiation process and a comparison of the two methods for generating EBs are provided in Figure 1. Additionally, the use of the spherical plate leads to a greatly increased efficiency in terms of yield, in addition to the incubator space, labor, and time required.

### Optimizing the seeding of iSAB-derived pacemaker cells

In extensive pilot experiments, we analyzed various dissociation enzymes and kits according to the manufactureŕs instructions (Supplementary Figure S1A). Thereby, Pierce™ Primary Cardiomyocyte Isolation Enzymes (hereafter referred to as PCIE) (Thermo Fisher Scientific; Waltham, USA) provided the most efficient dissociation, leading to the single cells required to generate a synchronized cell layer on MEA (Supplementary Figure S1B). Consequently, dissociation was performed using 50% PCIE dissolved in PBS for 30 min for all MEA analyses. Following this step, we optimized the number of cells seeded on the MEA, which ranged from 50,000–400,000 cells to finally ∼130.000–150.000 cells covering the electrode field (Supplementary Figure S1C).

Based on our previous investigations comparing different concentrations of Matrigel and gelatin (data not shown), Matrigel (1:50) was consistently used to coat the MEA for all experiments described herein.

### MEA measurements using the final differentiation protocol

Comparison of EB generation methods HD and SP in terms of drug-induced cell responses. In light of previous findings, hanging drops, with 3 days of hanging, and spherical plates with a duration of 2 days were directly compared utilizing substance testing on a microelectrode array. Simultaneously started differentiations were cultured until day 20/21, after which they underwent collagenase IV treatment followed by separation into iSABs. The following day after filtration (two days after collagenase treatment), iSABs were dissociated using PCIE. The enzyme solution was diluted by 50% with PBS and incubated for 30 min. The final cell suspension was then placed onto a pre-coated MEA array (Matrigel 1:50, 30 min) at a density of 130,000 cells per MEA in a fresh differentiation medium without ascorbic acid. The MEA arrays used were purchased from Multi-Channel-Systems (60MEA100/10iR-Ti and 60MEA200/30iR-Ti). The arrays were cultured under standard conditions for 10 days, with daily medium changes to facilitate the synchronization of the pacemaker cells. The last medium change was performed at least three hours before the measurement. On days 2, 5, and 10, the pacemaker cells generated by HD or SP were measured on MEA arrays to detect synchronization over time. For this purpose, MEA arrays were measured using the MEA2100 system from Multi-Channel Systems, with a 10 kHz sampling rate, temperature control set to 37°C, and ambient atmosphere. Following adaptation on the pre-warmed headstage, the field potential was measured. We calculated the mean heart rate, conduction velocity, amplitude, slope, and duration. We evaluated five electrodes per MEA for all parameters, except for duration (for which we evaluated ten electrodes) and velocity (which had only one value measured over the entire electrode field). Measurement and analysis were performed using the dedicated cardio-specific software, Cardio2D/ Cardi2D + software (Multi-Channel-Systems).

### Substance testing

The effects of test substances/drugs were analyzed using 1 M stock solutions of isoprenaline and carbachol that were prepared with ddH_2_O. ZD7288, which is also known as (4-(N-ethyl-N-phenylamino)-1,2-dimethyl-6-(methyl-amino) pyrimidinium chloride) was dissolved in DMSO to obtain a stock solution of 5 mM and 50 mM, respectively. For the measurement of concentrations between 10^−9^ and 10^−6^ (10^−4^), further dilutions were made in the differentiation medium. All substances and DMSO were purchased from Sigma Aldrich. After adaptation and a control measurement without substrate addition, increasing substrate concentrations were added gradually and washed in by gentle distribution using a pipette for 1 min. Substrate concentrations were added in small volumes to prevent the wash-in process from affecting the results. Subsequent measurements of the field potential was made for one minute.

To further investigate the time dependence of ZD7288-induced effects, cells were measured under baseline conditions and at intervals of 1, 2, 5, 10, and 20 min after the addition of 5 µM ZD7288, as used in Jung et al. (20). Finally, MEAs were then cleaned using TergazymA (Alconox Inc., White Plains, USA) (1% in ddH2O) overnight on a shaker and subjected to water rinsing and autoclaving.

### Immunostaining

Separated pacemaker cells were seeded on Matrigel-coated coverslips. After initial fixation of the cells with 4% PFA for 20 min, the cells were washed three times with PBS for 5 min each, followed by permeabilization (0.1% TritonX and 0.1 sodium citrate in PBS) for 3 min. After a first blocking was performed using a blocking solution (0.2% gelatin and 10% FCS in PBS), the cells were incubated in the dark for 1 h with the following primary antibodies diluted in antibody solution (0,01% Saponin, 0,2% gelatin, 10% FCS in PBS): αMHC [mouse anti- heavy chain cardiac myosin antibody (3–48)](Abcam Ab15) 1:200, α-sarcomeric actinin (rabbit anti—sarcomeric alpha actinin antibody)(Abcam ab68167) 1:100, HCN4 (rabbit anti-hyperpolarization-activated cyclic nucleotide-gated potassium channel 4 antibody)(Alomone Labs APC-052) 1:100, cardiac troponin (mouse anti-cardiac troponin t antibody)(Abcam ab8295) 1:150, Cx30.2 (rabbit anti-connexin 30.2 antibody)(Invitrogen 40–7,400) 1:50. Cells were washed with blocking solution, after which they were incubated for 45 min with the appropriate secondary antibody diluted in antibody solution: Alexa Fluor 647 (Donkey anti-mouse IgG H&L, Abcam 150111) 1:500, Alexa Fluor 594 (Goat anti-rabbit IgG H&L, Abcam 150080) 1:500, Alexa Fluor-488 Phalloidin (Invitrogen, A12379) 1:500, DAPI (1:2,000). Finally, the cells were washed with PBS for 5 min and embedded in FluorSave mounting medium (Merck, Burlington, USA). Microscopic imaging was performed using the ELYRA PS.1 LSM 780 microscope with a 40× or 63× oil objective.

### Statistical analysis

Statistical analysis was performed using GraphPad Prism. The diameter of the EBs was studied using a One-way ANOVA with Tukey's Multiple Comparison test. The number of beating foci was analyzed by One-way ANOVA with Bonferroni's Multiple Comparison Test, while the optically determined beating frequencies were analyzed by One-Way ANOVA with Tukey's Multiple Comparison Test. The MEA measurements of controls between HD and SP were compared using an unpaired *t*-test with Welch's correction. The effects of substrate administration were assessed using One-way ANOVA RM or Friedman's test with Dunnett's Multiple Comparison Test, after normality testing (Shapiro–Wilk Normality test). Data are presented as the mean ± SD. Single values are plotted, while Box plots indicate the mean and minimum/maximum whiskers. A *p*-value of *p ≤ 0.05, **p ≤ 0.01, and ***p ≤ 0.001 was considered significant.

## Results

Reliable and representative MEA measurements require a well-formed and interconnected cell layer on the electrode array. Our pluripotent stem cell-derived cardiac pacemaker cells are terminally differentiated and no longer proliferate, similar to other PSC-derived cardiomyocytes (41–43). Furthermore, cells within individual iSABs (clusters) exhibit strong cell-cell connections. In contrast, iSABs display limited formation of new connections to other iSABs. Therefore, to achieve multicellular syncytia of iSAB-derived pacemaker cells, we previously optimized iSAB dissociation to obtain single cells with subsequent seeding (e.g., for MEA analysis). Additionally, we ensured surface coating and appropriate cell numbers as seeding conditions to facilitate adherence and syncytium formation among our pacemaker cells. Exemplary images can be found in Supplementary Figure S1. In addition to light microscopic images of separated pacemaker cells, we performed immunostaining for essential contractile proteins such as α-sarcomeric actinin, cardiac troponin T, and α-myosin heavy chain as well as pacemaker cell markers HCN4 and Cx30.2 (Supplementary Figure S2) for further characterization.

We then focused on optimizing the generation of EBs to enable future standardized applications like substance testing. Therefore, we relied on the recently available *sphericalplates* 5D (Kugelmeiers).

### Process optimization during differentiation using a spherical plate for embryoid body generation instead of hanging drops

The formation of EBs is a commonly used initial stage in the differentiation of pluripotent stem cells into tissue-specific cell subtypes. Three-dimensional aggregation and organization of cells is a crucial step in the induction of differentiation. The HD technique, a frequently used approach for manual, small-scale production of EBs, is a technique that is time-consuming, requires significant space, and is cost-intensive, producing low output. To achieve large-scale production, a technique that generates a high number of EBs in a simplified manner is preferable. Recently, spherical multiwell plates (SP) (*Sphericalplate* 5D, Kugelmeiers, Switzerland) have emerged as a time- and space-saving alternative, that allows the production of size-standardized EBs on a large scale. Consequently, we evaluated and compared both methods, maintaining a slightly modified version of our established iSAB production protocol as a reference (20, 35).

**Figure 2:**
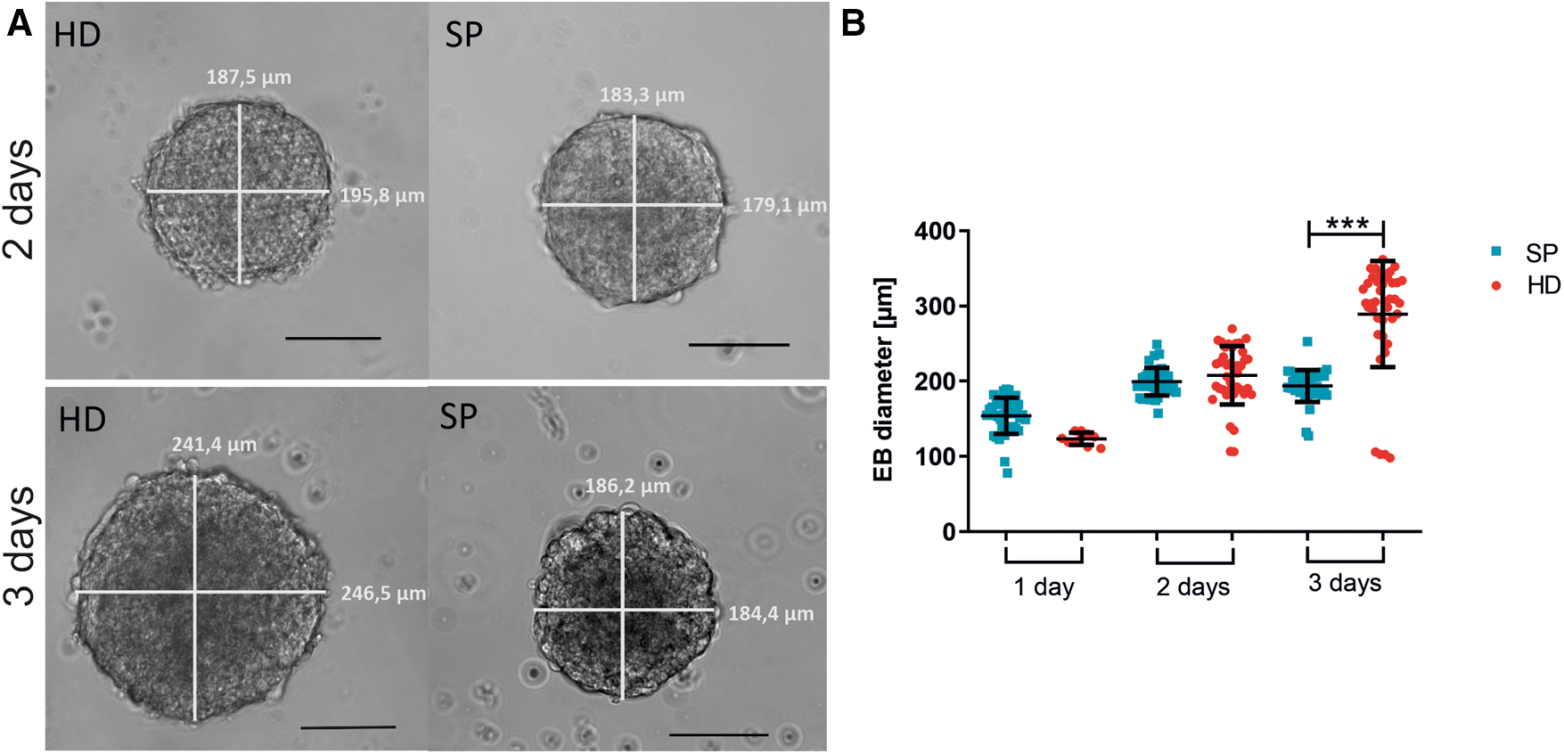
Embryoid body (EB) formation and diameter comparing hanging drop (HD) and spherical plate (SP) generation techniques. (**A**) Representative images of EBs generated by either HD or SP for two or three days. Scale bar 100 µm. (**B**) EB diameter is shown as the mean of the bi-directional (vertical and horizontal) measurement after generating EBs in HD (red) or SP (blue) for one, two, or three days (1 day–3 days) in 4 independent experiments. Data represent mean ± SD, as well as single values. *n* = 4, 10 EBs per condition measured; (***p < 0.001; One-way ANOVA with Tukey's Multiple Comparison Test).

In four independent experiments (each 10 EBs per condition), we analyzed the diameter of EBs after one, two, or three days of formation time (see Figure 2). Exemplary images of EBs are shown in Figure 2A. We found that EBs formed in SP exhibited greater consistency in size across multiple experiments than those formed in HD. Moreover, the collection of EBs in HD on day one was challenging due to their small size, with approximately 400 cells used per EB. The larger diameter of EBs, observed three days after the formation of HD can be attributed to the comparatively greater available space in comparison to SP. However, excessively large diameters are associated with longer diffusion paths, therefore potentially resulting in inferior nutrient supply to the centered cells (44). Even in individual experiments, there was considerable variation in the EB diameter in HD depending on the position on the plate. This could potentially lead to further inhomogeneity in subsequent experiments (Figure 2B).

**Figure 3:**
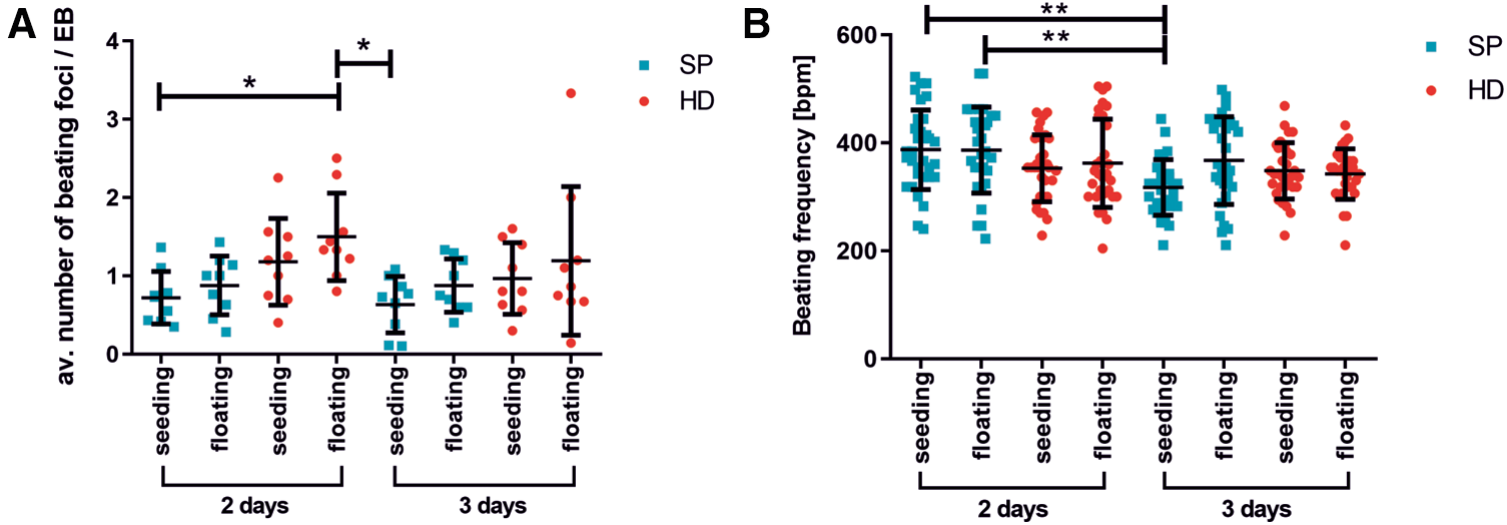
Beating activity of pacemaker cell clusters after generation with hanging drop or spherical plate technology. (**A**) average number of beating foci per EB calculated for 10 adherent EBs on days 16/17 of differentiation. The following conditions were analyzed: EB formation for 2 or 3 days with HD (red) or SP (blue), directly seeded or with an additional intermediate floating cultivation for 3–4 days. A total of 3 wells of 10 EBs were analyzed per condition. *n* = 3. Data represent mean ± SD, as well as single values; (*p < 0.05; One-Way ANOVA with Dunn's Multiple Comparison Test). (**B**) Beating frequency of iSABs (swimming pacemaker clusters) on days 25/28 of differentiation as a function of EB formation method, duration (2 days or 3 days), and following seeding. The same conditions as described in A) were tested. Beating frequency was measured on isolated iSABs on day 25/28 of culture. A total of 5-10 foci were analyzed per condition. *n* = 3. Data represent mean ± SD, as well as single values. (**0.001 ≤ *p < *0.01 One-Way ANOVA with Dunn's Multiple Comparison Test).

Moreover, the number of beating areas per EB was optically analyzed (Figure 3A). The EBs were generated using either the HD or SP method and then either directly plated onto gelatin-coated petri dishes or cultured as non-adherent EBs for an additional 3–4 days before plating. On days 16/17 of differentiation, the average number of beating foci per initial EB was determined. The use of HD for two days followed by floating culture resulted in a significant increase in the number of beating foci per EB, as compared to SP-generated EBs under seeding/floating culture conditions. Thereby, various differentiation time points were analyzed; Figure 3A displays the outcomes on days 16/17. Longer EB formation periods could lead to a lower number of beating foci, indicating further maturation and synchronization of the cells.

**Figure 4:**
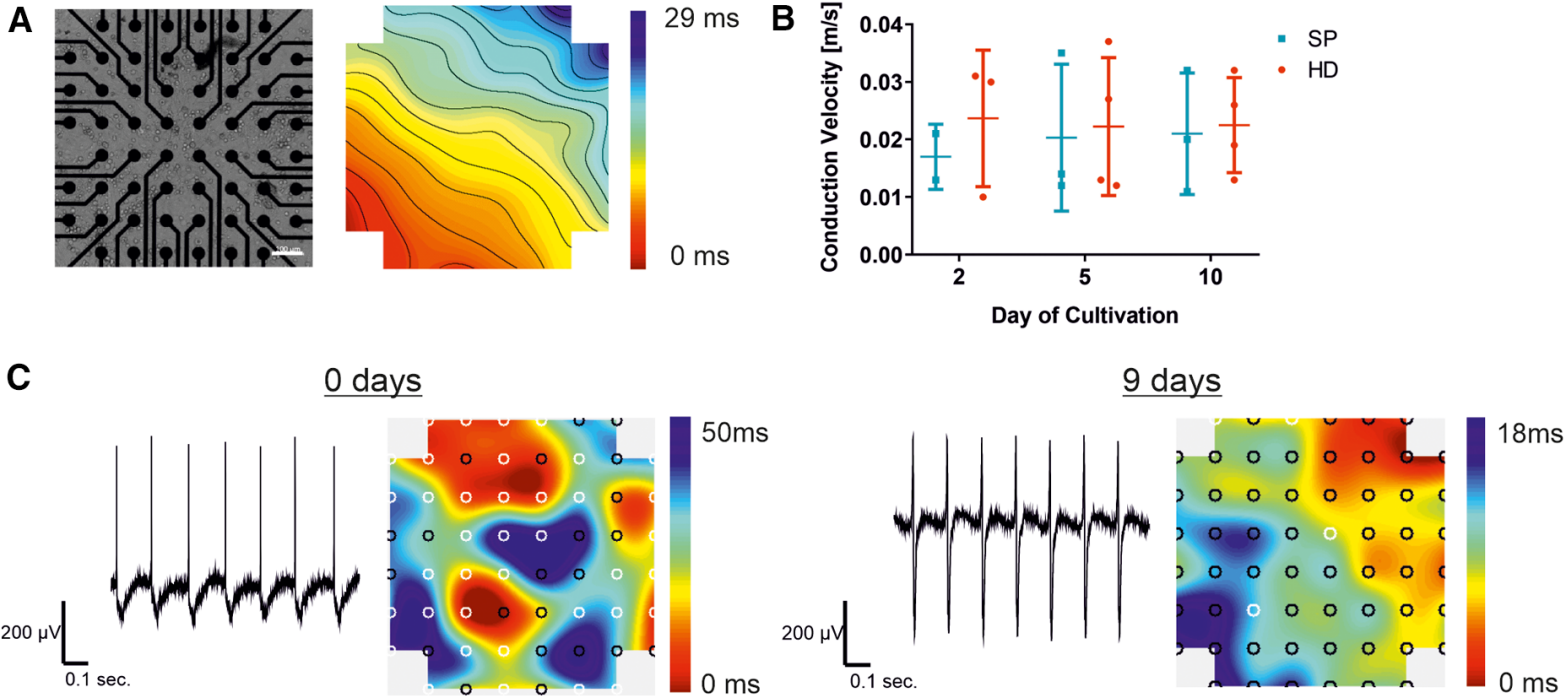
Establishment of a synchronized syncytium on MEA arrays. Analysis of pacemaker cell layers 2, 5, and 10 days after cell seeding on the MEA chip, comparing the EB generation technique to the hanging drop technique (HD; red) and spherical plate (SP; blue). (**A**) Example of a bright field microscopic control image of pacemaker cells cultured on the MEA chip and a corresponding color map of the measured field potential illustrating the excitation propagation measured as latency [ms] are displayed. Thereby, latencies between neighboring isochronal lines represent 1 ms. In the MEA system displayed, a latency of 1 ms in the vertical/horizontal direction of neighboring electrodes corresponds to a conduction velocity of 0.1 m/s (100er MEA), Scale bar 100 µm. (**B**) Development of the conduction velocity with increasing culture time, comparing HD (*n* = 4) and SP (*n* = 3). Data represent mean ± SD with single values. Significance was analyzed using a two-way ANOVA-RM with Tukey's Multiple Comparison Test). (**C**) Representative field potential traces of exemplary pacemaker cells on the day of cell seeding and after a further 9 days of culture, corresponding color maps show both increasing synchrony of the cell layer and signal propagation.

The beating frequency is a decisive parameter for functional cell characterization and therefore a crucial control for successful differentiation. In this regard, Figure 4B illustrates the beating frequencies of iSABs at days 25/28 of differentiation, depending on the type of EB formation method, duration, and subsequent culture technique. As the beating frequency of iSABs derived from 2-day SP-EB is comparable to or even slightly higher than that of those derived from HD-EB, resulting in approximately 400 bpm, which reflects the physiological range of the murine pacemaker, we integrated the SP method into our differentiation protocol. Furthermore, it is more convenient for the user and saves time, space, and plastic resources. For the subsequent drug testing experiments, we utilized SP-EB formation as it is a highly standardized method for generating EBs. This method was implemented for 2 days, and it yielded more promising results in terms of beating frequency and consistency when directly seeded onto gelatin-coated wells.

### Development and maturation of a synchronized syncytium

After establishing an optimized dissociation and re-seeding protocol, our objective was to determine a suitable time frame for drug testing experiments. This required the creation of an electrically coupled and synchronized cell layer with a stable conduction velocity. Therefore, cells were cultured for up to 10 days on the MEA chip. Microscopic control and field potential measurements were carried out on days 0, 2, 5, and 10 after seeding of iSAB-derived single cells to study the synchronization and to compare pacemaker cell layers from HD or SP-induced differentiation (Figure 4).

The color map, shown in Figure 4A), represents the cardiac activity at a distinct time point. The signal originates from a signal-generating area (pacemaker; red area) before propagating to the surrounding cells with a decent latency. The propagation time corresponds to the longest time span between the minimum and maximum points, while the lines illustrate areas of equal (isochronous) activation time. A video of the cell layer with the corresponding signal propagation can be found in the (Supplementary Movie S1). Intercellular connections enable the development of a cell layer, which can be seen in an exemplary bright-field microscopic picture of an MEA chip (Figure 4A). Conduction velocity, a key parameter in MEA analysis, was measured over a period of 10 days following cell seeding. No significant changes were detected with increasing culture time, as shown in Figure 3B. Single values demonstrate a smaller number of data points two days after seeding, compared to the later time points. Shortly after seeding the cells, the conduction velocity could only be determined in a small area because the cell layer showed different pacemaker foci and was not yet synchronized (Figure 4C). This reveals the need for maturation on the MEA chip. By prolonging the cultivation time, the measurable electrode regions can be expanded to cover the entire area for precise conduction velocity calculation. This results in a more accurate detection of the conduction velocity, increasing the amount of data points (days 5 and 10). In HD cell layers the velocity remained almost stable compared to SP, where a slight rise was observed between day 2 and day 5 of cultivation on MEA chips with a subsequent stabilization between 0.02 and 0.03 m/sec. Exemplary traces of measured field potentials on day 0, the day of cell seeding, and after 9 further days of culture on the MEA are shown in Figure 4C. The traces became more balanced between the maximum and minimum with increasing culture times. Signal propagation matures as indicated by shorter latencies, and the number of pacemaker foci decreases with processing synchrony, as shown in the corresponding color maps. From several pacemaker foci, one takes over the main activity, resulting in a transition from undirected to more directed signal propagation.

### Response to sympatho- and parasympathomimetics depending on the EB generation approach (HD vs. SP)

Finally, we aimed to validate our established protocols with respect to their suitability for testing novel drugs. We pursued this goal by verifying the physiological response of the cells to test substances. Electrophysiological measurements were performed on 60-electrode MEAs, including one reference electrode, after a 10-day maturation period on the MEA.

**Figure 5:**
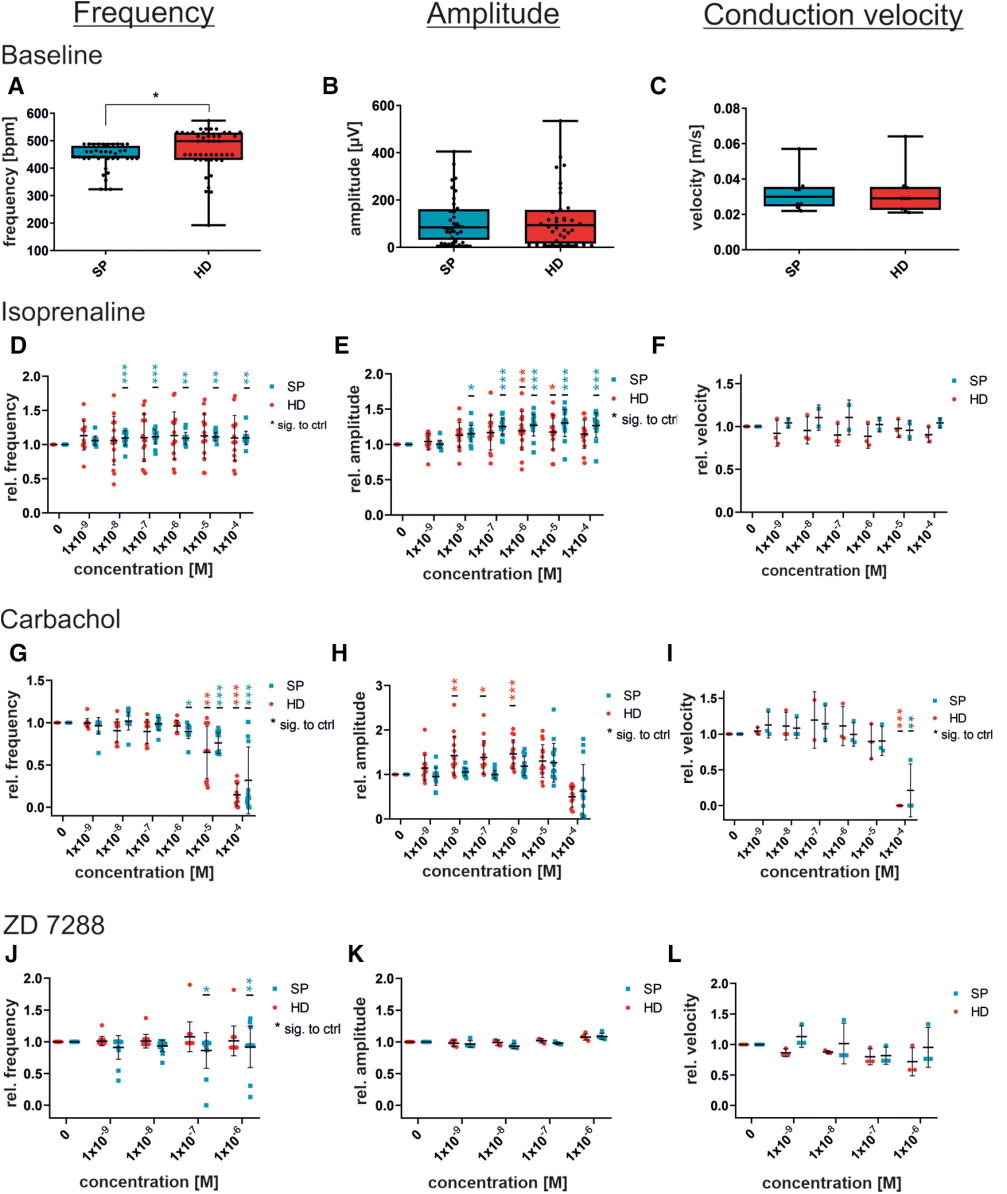
Concentration-dependent response to substances in iSAB pacemaker cells comparing the spherical plate (SP) and hanging drop (HD) techniques. Changes in the physiological parameters contraction frequency, amplitude, and conduction velocity before drug wash-in (baseline) (**A–C**) and after the administration of isoprenaline, a non-selective beta-sympathomimetic drug (**D–F**), carbachol, a parasympathomimetic drug (**G–I**) and ZD7288, a funny channel blocker (**J–L**) is illustrated. Red dots indicate HD-generated pacemaker cells and blue dots indicate SP-generated pacemaker cells. For control (baseline) measurements, a set of n = 9 MEAs (45 electrodes for frequency and amplitude) (**A–C**) was analyzed, whereas drug administration was measured at *n* = 15 electrodes from 3 MEAs for frequency and amplitude, and *n* = 3 MEAs each for velocity. Data were normalized to the respective untreated control (baseline). Box plots show single values and whiskers at minimum/maximum. Treatment graphs show mean ± SD as well as single values. For normality analysis, the Shapiro–Wilk Test was used. Significance was calculated using an unpaired *t*-test with Welch's correction for control (**A–C**). The significance of the respective untreated control was calculated using a one-way ANOVA RM or Friedman Test with Dunnett's Multiple Comparison Test (drug administration **D–L**). *p ≤ 0.05, **p ≤ 0.01, ***p ≤ 0.001.

Comparing the baseline parameters without substrate administration, the measurements revealed similar values for beating frequency, amplitude, and conduction velocity between the two EB generation strategies (Figures 5A–C). The data were obtained from all baseline measurements of the 9 MEAs before the drug wash-in. For beating frequency and amplitude, a total of 45 electrodes from 9 MEAs were analyzed. The displayed single values indicate low MEA to MEA variability. The mean contraction frequencies range from 450 to 550 bpm, while the conduction velocity reaches values around 003 m/s. Additionally, there is a relatively small average FP amplitude of approximately 80 µV.

Administration of isoprenaline caused an increase in beating frequency in response to β-adrenergic activation (Figure 5D). SP-generated cells displayed a significant elevation in beating frequency after being given 10 nM and increasing concentrations, whereas HD cells showed only a non-significant rise in beating frequency. Exemplary field potential traces, shown in Supplementary Figure S3A, display increasing frequencies. There was a significant increase in the field potential amplitude, with rising concentrations, observed in both SP and HD. Conversely, the differences in conduction velocity in response to isoprenaline were not significant. Moreover, Figure 5 illustrates the cellular response to the parasympathomimetic drug carbachol during MEA measurements. Administration of carbachol in the µM range resulted in the expected significant decrease in frequency and conduction velocity, independent of the EB generation process. The amplitude of HD-derived cells significantly increased with 0.01–1 µM carbachol, while changes in SP-derived cells were not significant. Notably, there were variations between HD and SP at concentrations of 0.01 and 0.1 µM carbachol. Representative field potential traces show a carbachol-induced reduction in beating frequency and an increase in amplitude (HD) (Supplementary Figure S3B). Although there were slight variations in the responses observed at individual concentrations of the tested substances between the compared methods, the overall response to the administration of isoprenaline and carbachol was comparable with respect to beating frequency, amplitude, and conduction velocity.

### Pacemaker-specific effects after administration of ZD7288

Additionally, the non-selective funny channel inhibitor ZD7288 was analyzed for specific pacemaker cell responses (Figures 5J–L). iSAB cells showed a significant dose- and time-dependent reduction in beating frequency for SP, as expected according to our previous work (20). FP measurements revealed a stable, but slightly decreasing beating frequency. The amplitude remained nearly constant after the addition of ZD7288, showing a high level of agreement between the SP and HD techniques. Conduction velocities did not differ significantly between the HD and SP techniques. Exemplary field potential traces before and after the addition of the funny channel inhibitor are shown in Supplementary Figure S3C.

**Figure 6:**
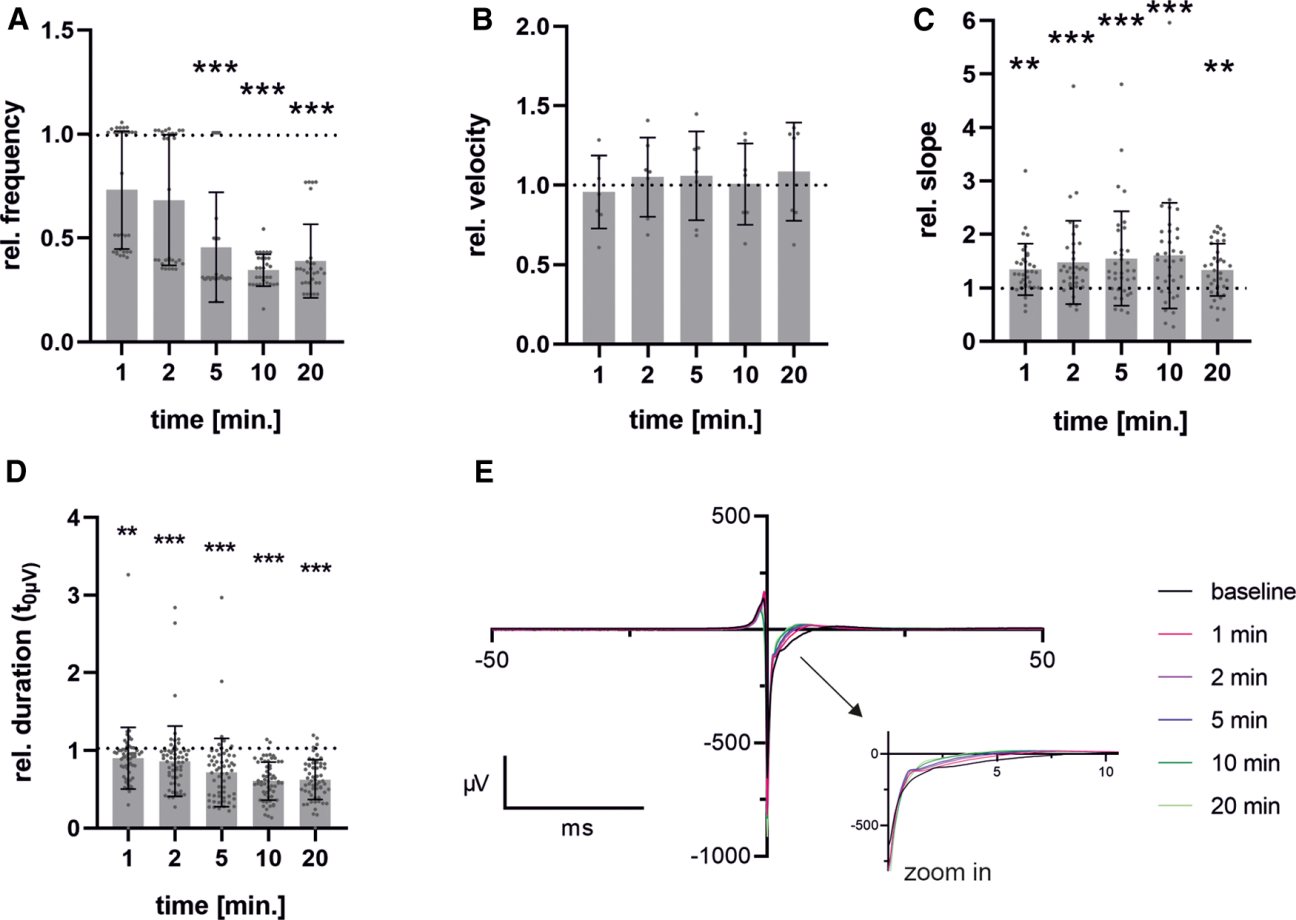
Time-dependent effects after administration of ZD7288. Pacemaker cells generated with spherical plate show a time-dependent response over a period of 1-20 min after administration of 5 µM ZD7288 on field potential beating frequency (**A**), conduction velocity (**B**), Na-peak slope (**C**) and duration from FP minimum to t_0µV_ (**D**) exemplary traces of time points after administration of ZD7288 are shown. To enhance clarity, the graph is truncated after 50 ms, with the critical section enlarged to improve visualization (**E**). Bar graph shows mean ± SD and single values. *n* = 6. Data were normalized to the baseline condition. For normality analysis, the Shapiro–Wilk Test was used. Significance was calculated using ANOVA RM or Friedman Test with Dunnett's Multiple Comparison Test. *p ≤ 0.05, **p ≤ 0.01, ***p ≤ 0.001.

Although there were slight differences observed when adding ZD7288 at different concentrations, the anticipated significant reduction in frequency was not seen. This may be due to either the concentration being too low or the measurement period being too short. Therefore, we performed the MEA measurements with the SP-generated cells and the previously published concentration of 5 µM [Jung et al. (20), Ca Imaging] to obtain a detailed view of the time-dependent response of pacemaker cells to the administration of ZD7288 (Figure 6). The field potentials of MEAs were measured at 1, 2, 5, 10 and 20 min after adding 5 µM ZD7288. A significant decrease in normalized beating frequency was observed at 5, 10, and 20 min compared to baseline conditions. Conduction velocity did not change significantly, but the peak slope increased significantly right after the addition of ZD7288 compared to baseline field potentials. Remarkably, we found that the second upstroke of the field potential, representing repolarization, was shortened with increasing application time, reaching the t_0µV_ point significantly faster. This could be seen as a pendant to the field potential duration usually measured in iPSC-CMs.

## Discussion

In this work, we introduced an established protocol for the separation, seeding, and cultivation of ESC-derived pacemaker cells on a MEA platform, leading to a synchronized cell layer of pacemaker cells. While the model was initially based solely on murine cells, our method allows for optimized cell separation and attachment, enabling standardized data collection and analysis, and overcomes the limitation of low cell numbers available isolated from explanted hearts.

In the past, other research groups have investigated hESC and iPSC-derived pacemaker-like cells by conducting electrophysiological characterizations of their differentiated cells. For instance, Mandel et al. extracted beating areas from early ESC/iPSC-derived EBs that covered only small regions of MEAs, and measured their field potentials using the respective few electrodes that yielded signals. Thereby, they reported heart rate variability and power-law behavior similar to that of the human sinoatrial node (21). Further characterization of the differentiated cells was not discussed, nor was the selection process, leaving it unclear whether these were truly pure subtypes or a mixture. Protze and colleagues demonstrated the electrophysiological responses of their hiPSC-derived SAN-Like Pacemaker Cells (SAN-LPC) after administration of isoprenaline and carbachol, as well as their antagonists, propranolol and atropine, using single-cell patch clamp analysis. In addition, they co-cultured cell layers of ventricular-like cardiomyocytes (VL-CM) with a small SAN-LPC aggregate seeded on top and performed MEA measurements. In this setting, VL-CM were successfully paced by the SAN-LPC aggregate (23). Schweizer et al. conducted comparable experiments and developed iPSC-derived pacemaker cell clusters that physiologically respond to isoprenaline, carbachol, and ivabradine on MEAs by co-culturing them with endoderm-like cells (24). Peischard et al. extended this system to analyze the effects of Coxsackievirus B3 on the pacemaker-specific expression of HCN4. The generated inducible cell line exhibited distinct beating rates between mature cells and those expressing CVB3 in comparison to the young pacemaker cell layers on MEAs (45). However, these setups do not offer insight into signal transduction within the pacemaker cell network. In a more complex system, Li et al. introduced Engineered Heart Tissues (EHTs) from subtype-specific cardiomyocytes. Before the generation of EHTs, this group analyzed drug responses in single-cell patch clamp assays. Subsequently, formed EHTs revealed substance-induced variability in beating frequencies as reflected by optically measured movements (*MUSCLEMOTION*) and signal propagation by optical mapping (28). In contrast to these approaches by other groups, we described a syncytium consisting of purified pacemaker cells on the MEA system to answer specific questions, including cellular synchronization and conduction velocity.

Prospectively, the ability to use highly enriched pacemaker cells for drug testing and cardiac risk assessment is of great importance, as various non-cardiac drugs have been shown, to induce sinus node dysfunction (46). Furthermore, it is possible to screen investigational drugs for their potential impact on the cardiac conduction system prior to approval. Early detection of increased cardiac risk could reduce the number of product withdrawals. In addition to improving the informative value of *in vitro* drug testing, this approach can also lead to a reduction in the number of ethically questionable and only partially transferable animal studies. Additionally, we have established the technological basis for disease modeling of pacemaker-specific diseases using the MEA system. This development will enable targeted risk assessment of patients with SAN dysfunction or disease. In this regard, our protocol may also be adaptable to induced human PSC-derived cells, resulting in high patient relevance.

The advantage of this analysis platform is the user-friendly and non-invasive electrophysiological analysis of cell layers. Developed approximately 40 years ago for the analysis of the electrical interaction of cells in tissues or single cells, the first MEA measurements were performed on cultured dorsal root ganglion neurons by Thomas et al. in 1972. The first measurements on myocytes followed shortly after (47, 48). Over the last decades, MEA technology has found a wide range of applications. The (high density) grid-like arrangement of the electrodes used in the experiment enables a high spatio-temporal resolution of the cell layers being measured. This allowed us to obtain information regarding intercellular connectivity and signal propagation, including latency and conduction velocity. In addition, individual parameters of the field potential, such as the peak amplitude or the Na-peak slope, can be analyzed in detail (37, 49). This method enables the analysis of electrophysiological questions in a cellular network, a more extended unit in comparison to, or as a complement to, patch-clamp measurements.

This study revealed the need for sufficient cell separation into single cells when aiming to generate a functional, synchronized cell layer. To accomplish the required cell density for MEA measurements, alternative methods of cell dissociation had to be optimized, as the previously used Accutase® (20) was only suitable for analyzing small numbers of viable cells, such as those used for by patch-clamp measurements.

The subsequent generation of a synchronized monolayer of beating cells, known as a syncytium, and the stabilization of beating and conduction velocity values require at least two to three days of culture. Conduction velocity values exhibit a slight increase during the initial days of culture before stabilizing after three to five days. This pattern mirrors the maturation of intercellular connections and indicates the time-dependent formation of a functional syncytium. Fahrenbach et al. described the development of a synchronized monolayer from hetero-cellular cultures comprising HL-1 cells and WT-fibroblasts after one day in culture (50), indicating differences between different cell (sub-)types.

As long as the basic parameters such as beating frequency and conduction velocity are stable, measurements can be performed between the 5th and 10th days of culture. However, attention should be paid to comparability by maintaining uniformity in the experimental design and signal analysis criteria. This may involve using a minimum number of “measurable” electrodes in addition to a minimum amplitude and beat rate. Under these conditions, it may be possible to conduct measurements over a longer period of time, up to several weeks. Therefore, it is important to consider CO_2_ control during long-term measurements and to implement automated seeding or continuous media flow to reduce shear stress, instead of relying on manual media changes.

The pacemaker activity in mice is able to generate beating frequencies in the range of 400–600 bpm (6.6–10 Hz). With our approach, the beating frequency of our stem cell-generated syncytium is at a constant rate, comparable to the intrinsic SAN activity *in vivo*. The syncytium also exhibits a conduction velocity of approximately 0.032 m/s, comparable to that of the mammalian SAN, thus confirming the suitability of iSABs as a surrogate for SAN function (51). For further investigation, we used isoprenaline and carbachol to examine the response to autonomic modulation. Sympathomimetic activation of iSABs results in an increased field potential amplitude and beating frequency, as observed in *in vivo* experiments. However, it has no effect on the conduction velocity. In contrast, titration of carbachol resulted in a significant reduction in beating frequency and conduction velocity. At 100 µM carbachol, the beating was almost abolished, while lower concentrations of carbachol resulted in an increased field potential amplitude (Figures 5G–I). Tertoolen et al. have shown a direct correlation between the upstroke velocity of an action potential and both the amplitude and the slope of the field potential (52). In a separate study, another research group investigated the effects of acetylcholine and noradrenalin on isolated rabbit sinoatrial myocytes and discovered an increase in AP upstroke velocity caused by the administration of acetylcholine (53). Taken together, the aforementioned results could explain the increase in field potential amplitude after the administration of carbachol. The parasympathomimetic activation of our cells with carbachol led to a decrease in the beating frequency, thus allowing a longer period of time for ion channel reactivation. Consequently, more channels are available, allowing for rapid depolarization in cases of action potential initiation, which results in an increased action potential upstroke velocity. To further verify this hypothesis, we used ZD7288, which is a potent inhibitor of HCN channels, to induce the pacemaker-specific “funny” current (I_f_) in SAN. In contrast to the selective inhibition of HCN by ZD7288, parasympathomimetic activation by carbachol induces broader effects on cell physiology. In addition to its ability to reduce automaticity by hyperpolarizing cells via muscarinic acetylcholine M2 receptor-stimulated K_Ach_ channels, carbachol can also reduce adenylyl cyclase (AC), thereby causing lower levels of intracellular cAMP, which affects both HCN channels and promotes dephosphorylation of calcium channels, the most prominent ion channel primarily responsible for depolarization in sinus nodal cells (54–57), caused by protein kinase A (PKA) inactivation.

Administration of ZD7288 resulted in a concentration- and time-dependent decrease in beating frequency but did not entirely abolish automaticity. Comparable significant effects on beating frequency were observed after a 5 min incubation period with 5 µM ZD7288, similar to those previously published for Ca-transients *in vitro* (20) and patch-clamp analysis of *ex vivo* preparations (58). These concentrations led to a reduction in beating frequency that reached a statistically significant level after 5–10 min (40%–50% of baseline). Higher concentrations, such as 30 or 100 µM, are expected to result in a more pronounced decrease (59–61), as additional ion channels may be unspecifically blocked (62, 63). The maintenance of automaticity could be explained by the fact that this mechanism may be based on both I_f_ (HCN) and I_CaL_ (Cav1.2, Cav1.3) (58, 64). Analysis of primary human pacemaker cells from the SAN has revealed further evidence for a combined mechanism based on the “membrane clock and calcium clock” (65).

A significant increase in the FP slope after the administration of ZD7288 is expected to correlate with a higher influx of Ca^2+^, partially compensating for the inhibition of the non-selective cation channel HCN. This increase in intracellular Ca^2+^ concentration, together with K^+^ efflux is expected to cause a shortening of the field potential duration, as shown by a reduced time span between the field potential reaching its minimum and a potential of 0 µV. This phase has been described as stage 1’–3’ of the field potential by Tertoolen et al. (52). We hypothesize that this parameter in pacemaker cells is an alternative to the field potential duration measured in hiPSC-CM. Due to the smaller field potentials and higher beating frequency, in addition to the absence of the resting membrane potential, it is not possible to detect the peak related to the field potential duration (FPD) or the QT interval in murine pacemaker cells.

Overall, microelectrode array analysis can provide reliable and cost-effective electrophysiological measurements that address both coherent cell layers and indirect effects on intercellular conduction and activation properties. Nevertheless, the direct correlation of field potential and action potential in iSABs can only be adequately achieved by additional patch-clamp recordings which are indispensable for the mechanistic understanding of electrophysiological processes, in particular the specific function of distinct channels and their respective currents. In conclusion, when comparing the two EB generation technologies, there was no significant difference observed between the two methods in terms of their physiological response to the tested substances. The slightly lower number of beating areas per SP-derived EBs compared to the HD technique can be tolerated due to the much higher throughput and lower workload of the SP technique. The advantages of these plates have also been used by other research groups, for example in ESC and MSC research (66, 67), but also in organ-specific tissue engineering (68–70).

## Conclusion

Overall, we introduced a protocol containing all necessary steps and meeting the requirements of highly demanding electrically active cells for a successful MEA measurement. Additionally, we presented spherical plates as a convenient and scalable method for the production of cardiac pacemaker cell cultures (iSABs). This approach enables the utilization of highly specialized pacemaker cells as a scalable and repeatable *in vitro* platform for drug and substance testing.

## Data Availability

The raw data supporting the conclusions of this article will be made available by the authors, without undue reservation.

## Supplementary material

Supplementary Figure S1 Optimization of enzymatic dissociation of iSAB clusters and subsequent cell density of pacemaker cells for MEA analysis. (**A**) Representative images of cluster dissociation using collagenase IV, Accutase, and Pierce Cardiomyocyte Isolation Enzyme (PCIE) according to the manufacturer's instructions compared to undissociated iSAB clusters. (**B**) Optimized dissociation with PCIE using an incubation time of 30 min and applying different enzyme concentrations (100, 75, 50, 25%). Adhesion and spreading of dissociated cells and clusters on Matrigel-coated coverslips are shown 4 and 18 days after enzymatic treatment. White circles show dead cells and debris, white arrows show coupling of cells. Scale bar: 100 µm. (**C**) Exemplary images of cell densities seeded after cluster dissociation ranging from 70,000 to 400,000 cells, with an optimal cell number of ∼130,000–150,000 cells. Scale bar: 100 µm.

Supplementary Figure S2 Contractile elements and pacemaker-specific marker proteins enable functionality in pacemaker cells. Separated pacemaker cells were positively stained for α-sarcomeric actinin, cardiac troponin t, and α-myosin heavy chain as contractile elements. HCN4 and connexin 30.2 are specifically expressed in pacemaker cells. Counterstaining was performed with DAPI for nuclei (blue). Scale bar 20 µm.

Supplementary Figure S3 Changes in field potentials in response to increasing concentrations of isoprenaline, carbachol, or ZD7288, comparing pacemaker cells generated with SP or HD Representative field potential traces were measured before and after the administration of isoprenaline (**A**), carbachol (**B**), and ZD7288 (**C**). Traces are shown for baseline and the concentration with the greatest difference from baseline for both EB generation methods; spherical plate (SP) on the left and hanging drop (HD) on the right side. Y-axis ticks are 100 and 200 µV, and x-axis ticks are 0.1 s

Supplementary Movie S1 Synchronized and functional cell layer of pacemaker cells on a MEA, related to Figure 4. Bright-field microscopic movie of synchronized pacemaker cells with the corresponding measurement of the field potential. The color map represents the field potentials (µV) for a defined period of time, with the individual colors indicating the differences in field potential. To improve the visibility of the signal propagation in the color map, this video section has a reduced frame rate.
